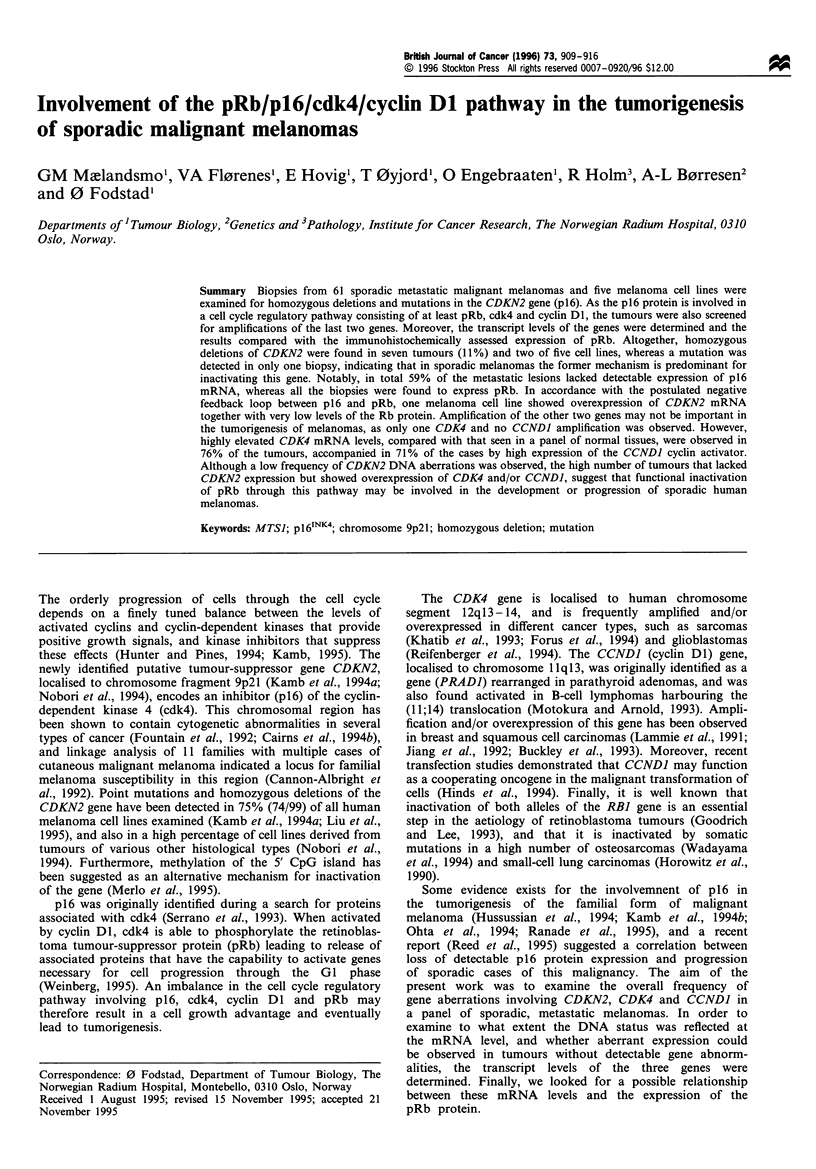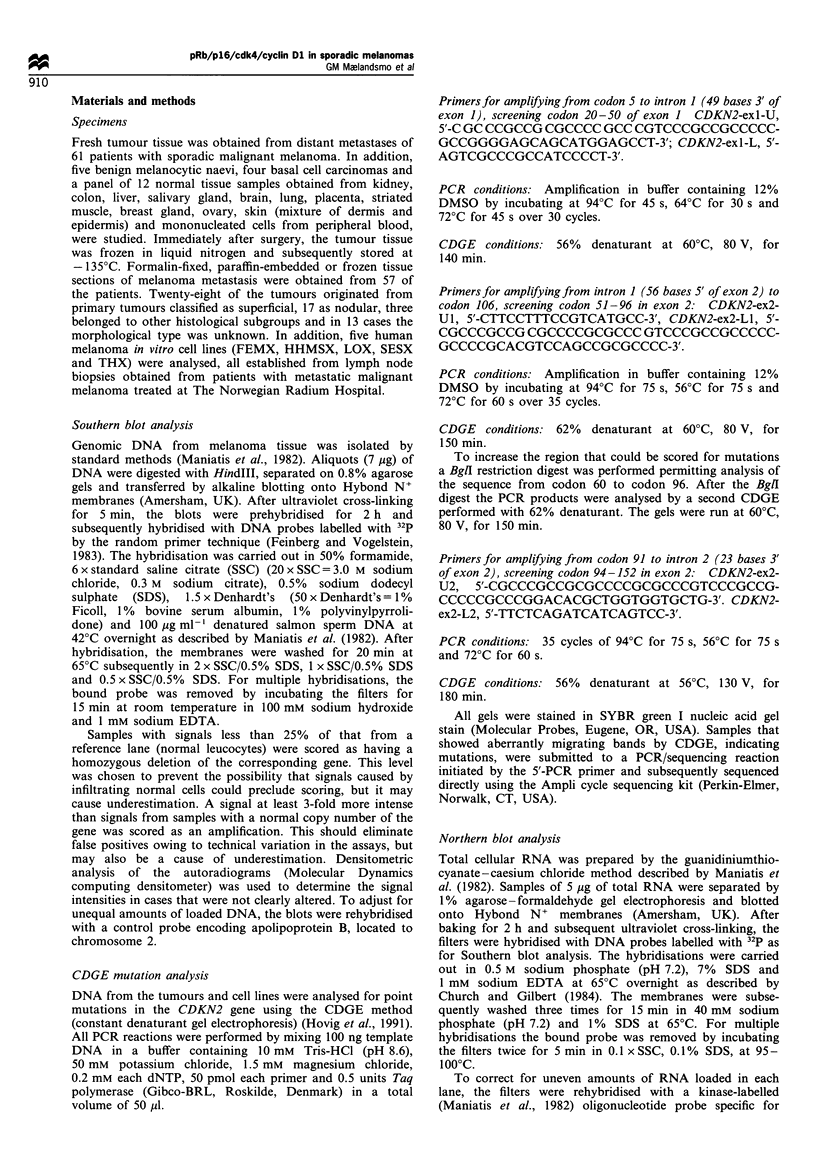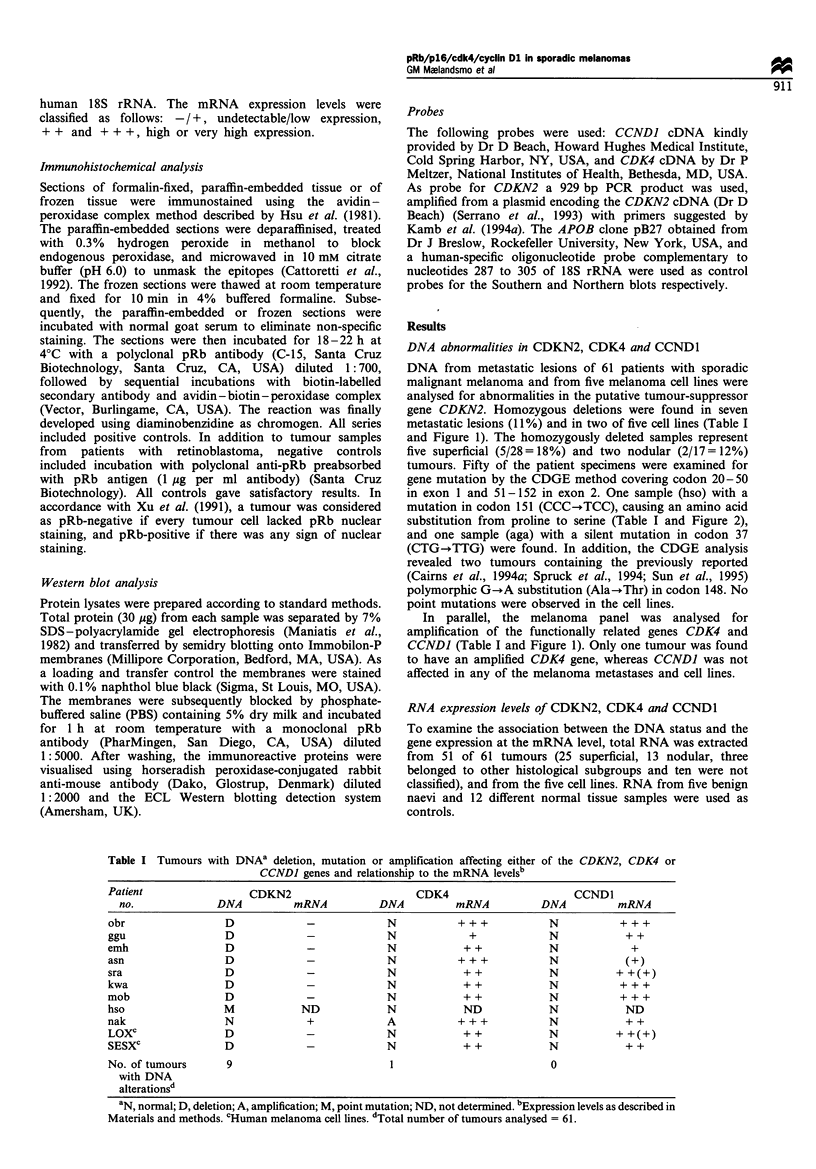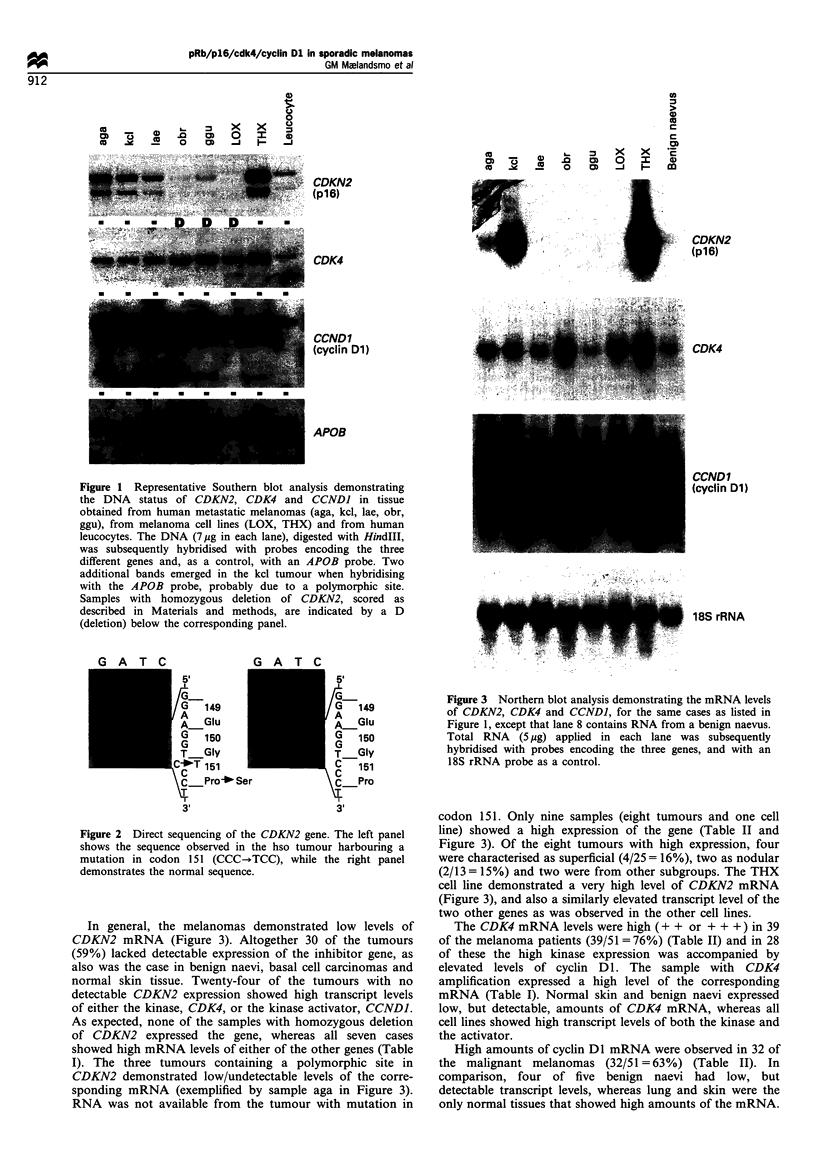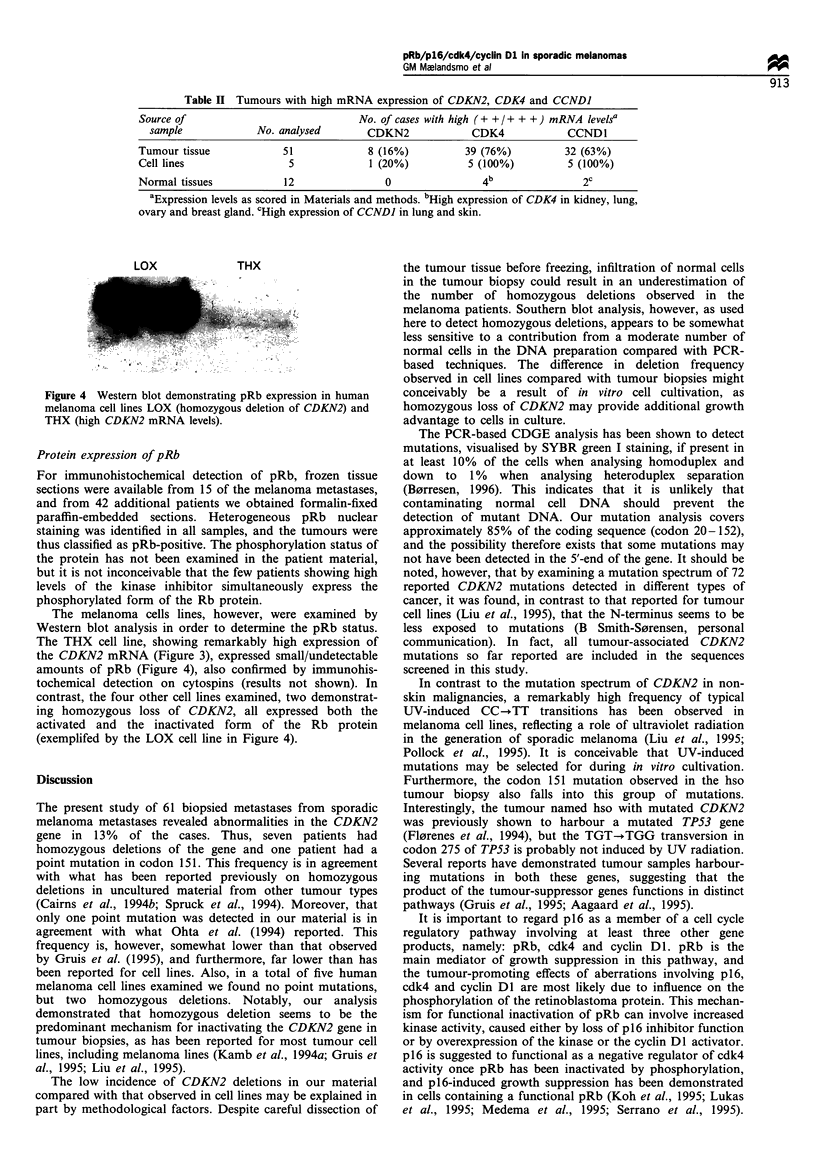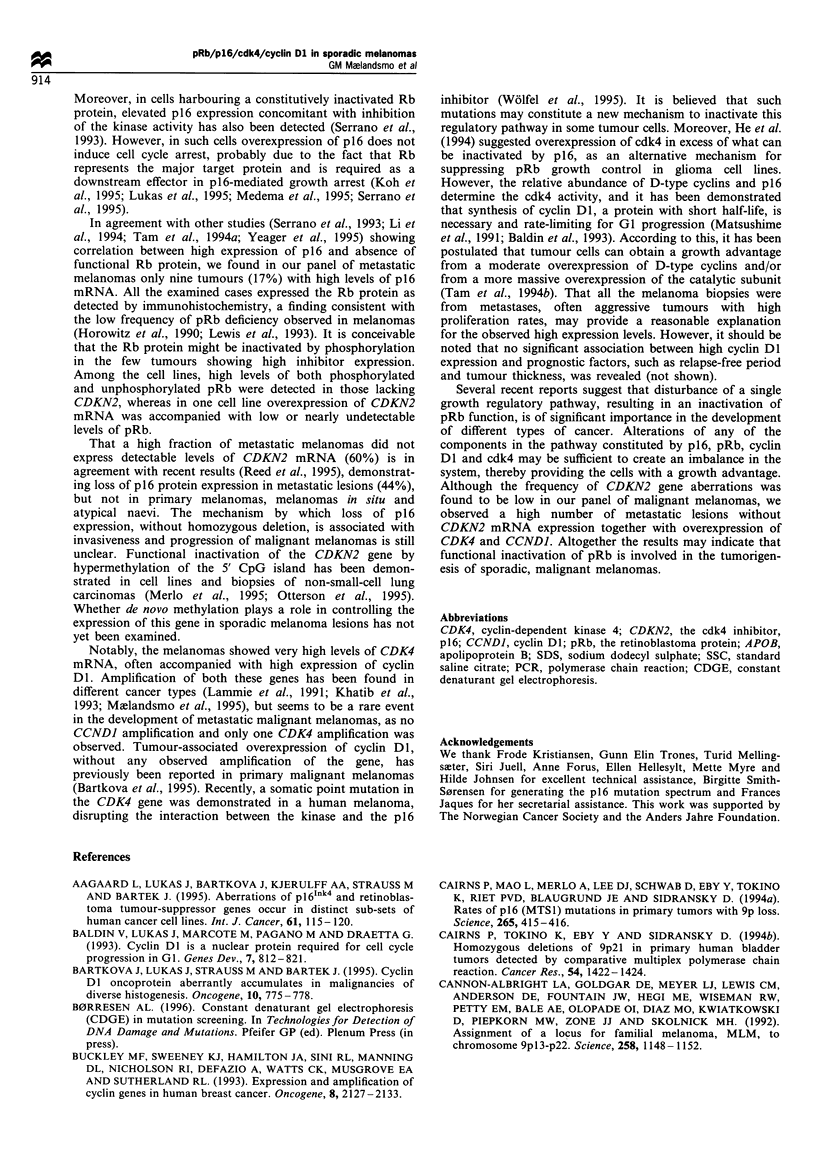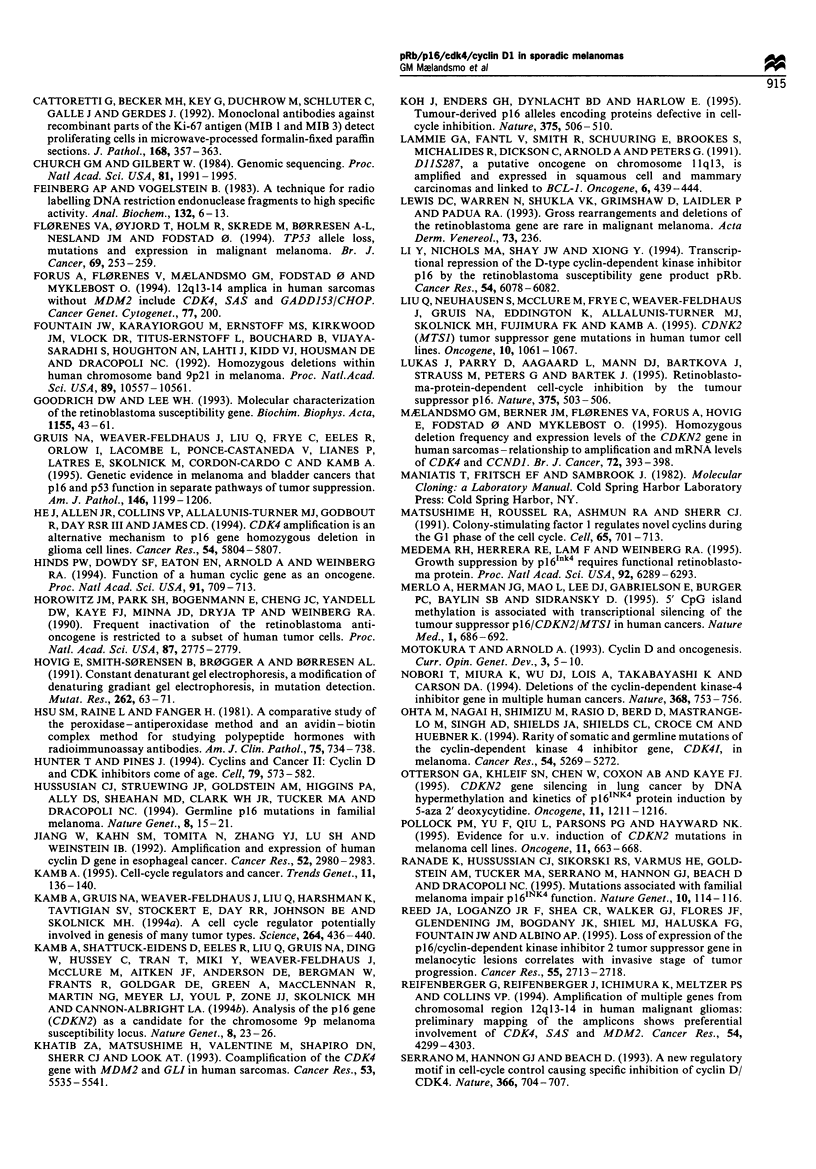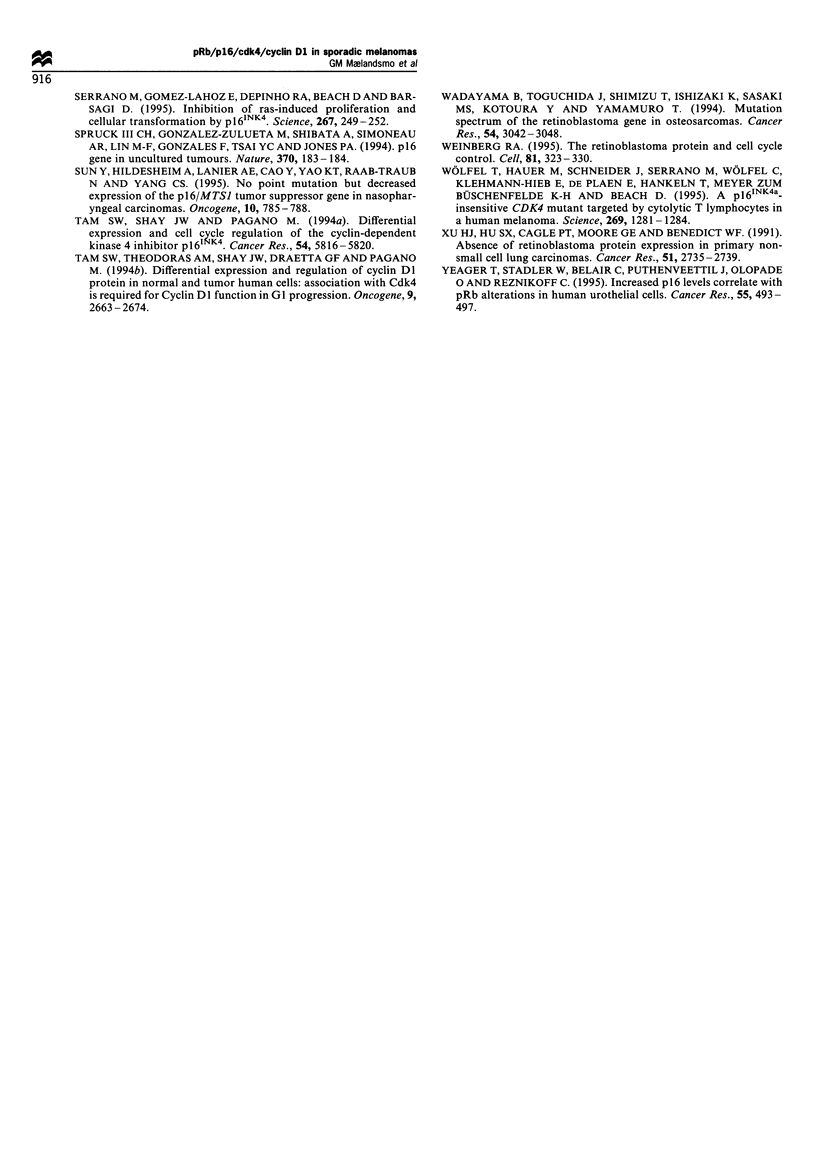# Involvement of the pRb/p16/cdk4/cyclin D1 pathway in the tumorigenesis of sporadic malignant melanomas.

**DOI:** 10.1038/bjc.1996.181

**Published:** 1996-04

**Authors:** G. M. Maelandsmo, V. A. Flørenes, E. Hovig, T. Oyjord, O. Engebraaten, R. Holm, A. L. Børresen, O. Fodstad

**Affiliations:** Department of Tumour Biology, Institute for Cancer Research, Norwegian Radium Hospital, Oslo, Norway.

## Abstract

**Images:**


British Journal of Cancer (1996) 73, 909-916
© 1996 Stockton Press All rights reserved 0007-0920/96 $12.00

### Involvement of the pRb/p16/cdk4/cyclin D1 pathway in the tumorigenesis of sporadic malignant melanomas

GM Mælandsmo[1], VA Flørenes[1], E Hovig[1], T Øyjord[1], O Engebraaten[1], R Holm[3], A-L Børresen[2] and Ø Fodstad[1]

*Departments of [1]Tumour Biology, [2]Genetics and [3]Pathology, Institute for Cancer Research, The Norwegian Radium Hospital, 0310 Oslo, Norway.*

**Summary** Biopsies from 61 sporadic metastatic malignant melanomas and five melanoma cell lines were examined for homozygous deletions and mutations in the *CDKN2* gene (p16). As the p16 protein is involved in a cell cycle regulatory pathway consisting of at least pRb, cdk4 and cyclin D1, the tumours were also screened for amplifications of the last two genes. Moreover, the transcript levels of the genes were determined and the results compared with the immunohistochemically assessed expression of pRb. Altogether, homozygous deletions of *CDKN2* were found in seven tumours (11%) and two of five cell lines, whereas a mutation was detected in only one biopsy, indicating that in sporadic melanomas the former mechanism is predominant for inactivating this gene. Notably, in total 59% of the metastatic lesions lacked detectable expression of p16 mRNA, whereas all the biopsies were found to express pRb. In accordance with the postulated negative feedback loop between p16 and pRb, one melanoma cell line showed overexpression of *CDKN2* mRNA together with very low levels of the Rb protein. Amplification of the other two genes may not be important in the tumorigenesis of melanomas, as only one *CDK4* and no *CCND1* amplification was observed. However, highly elevated *CDK4* mRNA levels, compared with that seen in a panel of normal tissues, were observed in 76% of the tumours, accompanied in 71% of the cases by high expression of the *CCND1* cyclin activator. Although a low frequency of *CDKN2* DNA aberrations was observed, the high number of tumours that lacked *CDKN2* expression but showed overexpression of *CDK4* and/or *CCND1*, suggest that functional inactivation of pRb through this pathway may be involved in the development or progression of sporadic human melanomas.

**Keywords:** *MTS1*; p16$^{INK4}$; chromosome 9p21; homozygous deletion; mutation

The orderly progression of cells through the cell cycle depends on a finely tuned balance between the levels of activated cyclins and cyclin-dependent kinases that provide positive growth signals, and kinase inhibitors that suppress these effects (Hunter and Pines, 1994; Kamb, 1995). The newly identified putative tumour-suppressor gene *CDKN2*, localised to chromosome fragment 9p21 (Kamb *et al.*, 1994*a*; Nobori *et al.*, 1994), encodes an inhibitor (p16) of the cyclin-dependent kinase 4 (cdk4). This chromosomal region has been shown to contain cytogenetic abnormalities in several types of cancer (Fountain *et al.*, 1992; Cairns *et al.*, 1994*b*), and linkage analysis of 11 families with multiple cases of cutaneous malignant melanoma indicated a locus for familial melanoma susceptibility in this region (Cannon-Albright *et al.*, 1992). Point mutations and homozygous deletions of the *CDKN2* gene have been detected in 75% (74/99) of all human melanoma cell lines examined (Kamb *et al.*, 1994*a*; Liu *et al.*, 1995), and also in a high percentage of cell lines derived from tumours of various other histological types (Nobori *et al.*, 1994). Furthermore, methylation of the 5′ CpG island has been suggested as an alternative mechanism for inactivation of the gene (Merlo *et al.*, 1995).

p16 was originally identified during a search for proteins associated with cdk4 (Serrano *et al.*, 1993). When activated by cyclin D1, cdk4 is able to phosphorylate the retinoblastoma tumour-suppressor protein (pRb) leading to release of associated proteins that have the capability to activate genes necessary for cell progression through the G1 phase (Weinberg, 1995). An imbalance in the cell cycle regulatory pathway involving p16, cdk4, cyclin D1 and pRb may therefore result in a cell growth advantage and eventually lead to tumorigenesis.

Correspondence: Ø Fodstad, Department of Tumour Biology, The Norwegian Radium Hospital, Montebello, 0310 Oslo, Norway
Received 1 August 1995; revised 15 November 1995; accepted 21 November 1995

The *CDK4* gene is localised to human chromosome segment 12q13–14, and is frequently amplified and/or overexpressed in different cancer types, such as sarcomas (Khatib *et al.*, 1993; Forus *et al.*, 1994) and glioblastomas (Reifenberger *et al.*, 1994). The *CCND1* (cyclin D1) gene, localised to chromosome 11q13, was originally identified as a gene (*PRAD1*) rearranged in parathyroid adenomas, and was also found activated in B-cell lymphomas harbouring the (11;14) translocation (Motokura and Arnold, 1993). Amplification and/or overexpression of this gene has been observed in breast and squamous cell carcinomas (Lammie *et al.*, 1991; Jiang *et al.*, 1992; Buckley *et al.*, 1993). Moreover, recent transfection studies demonstrated that *CCND1* may function as a cooperating oncogene in the malignant transformation of cells (Hinds *et al.*, 1994). Finally, it is well known that inactivation of both alleles of the *RB1* gene is an essential step in the aetiology of retinoblastoma tumours (Goodrich and Lee, 1993), and that it is inactivated by somatic mutations in a high number of osteosarcomas (Wadayama *et al.*, 1994) and small-cell lung carcinomas (Horowitz *et al.*, 1990).

Some evidence exists for the involvemnent of p16 in the tumorigenesis of the familial form of malignant melanoma (Hussussian *et al.*, 1994; Kamb *et al.*, 1994*b*; Ohta *et al.*, 1994; Ranade *et al.*, 1995), and a recent report (Reed *et al.*, 1995) suggested a correlation between loss of detectable p16 protein expression and progression of sporadic cases of this malignancy. The aim of the present work was to examine the overall frequency of gene aberrations involving *CDKN2*, *CDK4* and *CCND1* in a panel of sporadic, metastatic melanomas. In order to examine to what extent the DNA status was reflected at the mRNA level, and whether aberrant expression could be observed in tumours without detectable gene abnormalities, the transcript levels of the three genes were determined. Finally, we looked for a possible relationship between these mRNA levels and the expression of the pRb protein.

### Materials and methods

### Specimens

Fresh tumour tissue was obtained from distant metastases of 61 patients with sporadic malignant melanoma. In addition, five benign melanocytic naevi, four basal cell carcinomas and a panel of 12 normal tissue samples obtained from kidney, colon, liver, salivary gland, brain, lung, placenta, striated muscle, breast gland, ovary, skin (mixture of dermis and epidermis) and mononucleated cells from peripheral blood, were studied. Immediately after surgery, the tumour tissue was frozen in liquid nitrogen and subsequently stored at −135°C. Formalin-fixed, paraffin-embedded or frozen tissue sections of melanoma metastasis were obtained from 57 of the patients. Twenty-eight of the tumours originated from primary tumours classified as superficial, 17 as nodular, three belonged to other histological subgroups and in 13 cases the morphological type was unknown. In addition, five human melanoma *in vitro* cell lines (FEMX, HHMSX, LOX, SESX and THX) were analysed, all established from lymph node biopsies obtained from patients with metastatic malignant melanoma treated at The Norwegian Radium Hospital.

### Southern blot analysis

Genomic DNA from melanoma tissue was isolated by standard methods (Maniatis *et al.*, 1982). Aliquots (7 μg) of DNA were digested with *Hin*dIII, separated on 0.8% agarose gels and transferred by alkaline blotting onto Hybond $N^+$ membranes (Amersham, UK). After ultraviolet cross-linking for 5 min, the blots were prehybridised for 2 h and subsequently hybridised with DNA probes labelled with $^{32}P$ by the random primer technique (Feinberg and Vogelstein, 1983). The hybridisation was carried out in 50% formamide, 6 × standard saline citrate (SSC) (20 × SSC = 3.0 M sodium chloride, 0.3 M sodium citrate), 0.5% sodium dodecyl sulphate (SDS), 1.5 × Denhardt's (50 × Denhardt's = 1% Ficoll, 1% bovine serum albumin, 1% polyvinylpyrrolidone) and 100 $\mu g\ ml^{-1}$ denatured salmon sperm DNA at 42°C overnight as described by Maniatis *et al.* (1982). After hybridisation, the membranes were washed for 20 min at 65°C subsequently in 2 × SSC/0.5% SDS, 1 × SSC/0.5% SDS and 0.5 × SSC/0.5% SDS. For multiple hybridisations, the bound probe was removed by incubating the filters for 15 min at room temperature in 100 mM sodium hydroxide and 1 mM sodium EDTA.

Samples with signals less than 25% of that from a reference lane (normal leucocytes) were scored as having a homozygous deletion of the corresponding gene. This level was chosen to prevent the possibility that signals caused by infiltrating normal cells could preclude scoring, but it may cause underestimation. A signal at least 3-fold more intense than signals from samples with a normal copy number of the gene was scored as an amplification. This should eliminate false positives owing to technical variation in the assays, but may also be a cause of underestimation. Densitometric analysis of the autoradiograms (Molecular Dynamics computing densitometer) was used to determine the signal intensities in cases that were not clearly altered. To adjust for unequal amounts of loaded DNA, the blots were rehybridised with a control probe encoding apolipoprotein B, located to chromosome 2.

### CDGE mutation analysis

DNA from the tumours and cell lines were analysed for point mutations in the *CDKN2* gene using the CDGE method (constant denaturant gel electrophoresis) (Hovig *et al.*, 1991). All PCR reactions were performed by mixing 100 ng template DNA in a buffer containing 10 mM Tris-HCl (pH 8.6), 50 mM potassium chloride, 1.5 mM magnesium chloride, 0.2 mM each dNTP, 50 pmol each primer and 0.5 units *Taq* polymerase (Gibco-BRL, Roskilde, Denmark) in a total volume of 50 μl.

*Primers for amplifying from codon 5 to intron 1 (49 bases 3′ of exon 1), screening codon 20–50 of exon 1* *CDKN2*-ex1-U, 5′-C GC CCGCCG CGCCCC GCC CGTCCCGCCGCCCCC-GCCGGGGAGCAGCATGGAGCCT-3′; *CDKN2*-ex1-L, 5′-AGTCGCCCGCCATCCCCT-3′.

*PCR conditions:* Amplification in buffer containing 12% DMSO by incubating at 94°C for 45 s, 64°C for 30 s and 72°C for 45 s over 30 cycles.

*CDGE conditions:* 56% denaturant at 60°C, 80 V, for 140 min.

*Primers for amplifying from intron 1 (56 bases 5′ of exon 2) to codon 106, screening codon 51–96 in exon 2:* *CDKN2*-ex2-U1, 5′-CTTCCTTTCCGTCATGCC-3′, *CDKN2*-ex2-L1, 5′-CGCCCGCCG CGCCCCGCGCCC GTCCCGCCGCCCCC-GCCCCGCACGTCCAGCCGCGCCCC-3′.

*PCR conditions:* Amplification in buffer containing 12% DMSO by incubating at 94°C for 75 s, 56°C for 75 s and 72°C for 60 s over 35 cycles.

*CDGE conditions:* 62% denaturant at 60°C, 80 V, for 150 min.

To increase the region that could be scored for mutations a *Bgl*I restriction digest was performed permitting analysis of the sequence from codon 60 to codon 96. After the *Bgl*I digest the PCR products were analysed by a second CDGE performed with 62% denaturant. The gels were run at 60°C, 80 V, for 150 min.

*Primers for amplifying from codon 91 to intron 2 (23 bases 3′ of exon 2), screening codon 94–152 in exon 2:* *CDKN2*-ex2-U2, 5′-CGCCCGCCGCGCCCCGCGCCCGTCCCGCCG-CCCCCGCCCGGACACGCTGGTGGTGCTG-3′. *CDKN2*-ex2-L2, 5′-TTCTCAGATCATCAGTCC-3′.

*PCR conditions:* 35 cycles of 94°C for 75 s, 56°C for 75 s and 72°C for 60 s.

*CDGE conditions:* 56% denaturant at 56°C, 130 V, for 180 min.

All gels were stained in SYBR green I nucleic acid gel stain (Molecular Probes, Eugene, OR, USA). Samples that showed aberrantly migrating bands by CDGE, indicating mutations, were submitted to a PCR/sequencing reaction initiated by the 5′-PCR primer and subsequently sequenced directly using the Ampli cycle sequencing kit (Perkin-Elmer, Norwalk, CT, USA).

### Northern blot analysis

Total cellular RNA was prepared by the guanidiniumthiocyanate–caesium chloride method described by Maniatis *et al.* (1982). Samples of 5 μg of total RNA were separated by 1% agarose–formaldehyde gel electrophoresis and blotted onto Hybond $N^+$ membranes (Amersham, UK). After baking for 2 h and subsequent ultraviolet cross-linking, the filters were hybridised with DNA probes labelled with $^{32}P$ as for Southern blot analysis. The hybridisations were carried out in 0.5 M sodium phosphate (pH 7.2), 7% SDS and 1 mM sodium EDTA at 65°C overnight as described by Church and Gilbert (1984). The membranes were subsequently washed three times for 15 min in 40 mM sodium phosphate (pH 7.2) and 1% SDS at 65°C. For multiple hybridisations the bound probe was removed by incubating the filters twice for 5 min in 0.1 × SSC, 0.1% SDS, at 95–100°C.

To correct for uneven amounts of RNA loaded in each lane, the filters were rehybridised with a kinase-labelled (Maniatis *et al.*, 1982) oligonucleotide probe specific for human 18S rRNA. The mRNA expression levels were classified as follows: −/+, undetectable/low expression, + + and + + +, high or very high expression.

*Immunohistochemical analysis*

Sections of formalin-fixed, paraffin-embedded tissue or of frozen tissue were immunostained using the avidin−peroxidase complex method described by Hsu *et al.* (1981). The paraffin-embedded sections were deparaffinised, treated with 0.3% hydrogen peroxide in methanol to block endogenous peroxidase, and microwaved in 10 mM citrate buffer (pH 6.0) to unmask the epitopes (Cattoretti *et al.*, 1992). The frozen sections were thawed at room temperature and fixed for 10 min in 4% buffered formaline. Subsequently, the paraffin-embedded or frozen sections were incubated with normal goat serum to eliminate non-specific staining. The sections were then incubated for 18−22 h at 4°C with a polyclonal pRb antibody (C-15, Santa Cruz Biotechnology, Santa Cruz, CA, USA) diluted 1:700, followed by sequential incubations with biotin-labelled secondary antibody and avidin−biotin−peroxidase complex (Vector, Burlingame, CA, USA). The reaction was finally developed using diaminobenzidine as chromogen. All series included positive controls. In addition to tumour samples from patients with retinoblastoma, negative controls included incubation with polyclonal anti-pRb preabsorbed with pRb antigen (1 μg per ml antibody) (Santa Cruz Biotechnology). All controls gave satisfactory results. In accordance with Xu *et al.* (1991), a tumour was considered as pRb-negative if every tumour cell lacked pRb nuclear staining, and pRb-positive if there was any sign of nuclear staining.

*Western blot analysis*

Protein lysates were prepared according to standard methods. Total protein (30 μg) from each sample was separated by 7% SDS−polyacrylamide gel electrophoresis (Maniatis *et al.*, 1982) and transferred by semidry blotting onto Immobilon-P membranes (Millipore Corporation, Bedford, MA, USA). As a loading and transfer control the membranes were stained with 0.1% naphthol blue black (Sigma, St Louis, MO, USA). The membranes were subsequently blocked by phosphate-buffered saline (PBS) containing 5% dry milk and incubated for 1 h at room temperature with a monoclonal pRb antibody (PharMingen, San Diego, CA, USA) diluted 1:5000. After washing, the immunoreactive proteins were visualised using horseradish peroxidase-conjugated rabbit anti-mouse antibody (Dako, Glostrup, Denmark) diluted 1:2000 and the ECL Western blotting detection system (Amersham, UK).

*Probes*

The following probes were used: *CCND1* cDNA kindly provided by Dr D Beach, Howard Hughes Medical Institute, Cold Spring Harbor, NY, USA, and *CDK4* cDNA by Dr P Meltzer, National Institutes of Health, Bethesda, MD, USA. As probe for *CDKN2* a 929 bp PCR product was used, amplified from a plasmid encoding the *CDKN2* cDNA (Dr D Beach) (Serrano *et al.*, 1993) with primers suggested by Kamb *et al.* (1994*a*). The *APOB* clone pB27 obtained from Dr J Breslow, Rockefeller University, New York, USA, and a human-specific oligonucleotide probe complementary to nucleotides 287 to 305 of 18S rRNA were used as control probes for the Southern and Northern blots respectively.

### Results

*DNA abnormalities in* CDKN2, CDK4 *and* CCND1

DNA from metastatic lesions of 61 patients with sporadic malignant melanoma and from five melanoma cell lines were analysed for abnormalities in the putative tumour-suppressor gene *CDKN2*. Homozygous deletions were found in seven metastatic lesions (11%) and in two of five cell lines (Table I and Figure 1). The homozygously deleted samples represent five superficial (5/28 = 18%) and two nodular (2/17 = 12%) tumours. Fifty of the patient specimens were examined for gene mutation by the CDGE method covering codon 20−50 in exon 1 and 51−152 in exon 2. One sample (hso) with a mutation in codon 151 (CCC→TCC), causing an amino acid substitution from proline to serine (Table I and Figure 2), and one sample (aga) with a silent mutation in codon 37 (CTG→TTG) were found. In addition, the CDGE analysis revealed two tumours containing the previously reported (Cairns *et al.*, 1994*a*; Spruck *et al.*, 1994; Sun *et al.*, 1995) polymorphic G→A substitution (Ala→Thr) in codon 148. No point mutations were observed in the cell lines.

In parallel, the melanoma panel was analysed for amplification of the functionally related genes *CDK4* and *CCND1* (Table I and Figure 1). Only one tumour was found to have an amplified *CDK4* gene, whereas *CCND1* was not affected in any of the melanoma metastases and cell lines.

*RNA expression levels of* CDKN2, CDK4 *and* CCND1

To examine the association between the DNA status and the gene expression at the mRNA level, total RNA was extracted from 51 of 61 tumours (25 superficial, 13 nodular, three belonged to other histological subgroups and ten were not classified), and from the five cell lines. RNA from five benign naevi and 12 different normal tissue samples were used as controls.

**Table I** Tumours with DNA[a] deletion, mutation or amplification affecting either of the *CDKN2*, *CDK4* or *CCND1* genes and relationship to the mRNA levels[b]

| *Patient no.* | CDKN2 *DNA* | CDKN2 *mRNA* | CDK4 *DNA* | CDK4 *mRNA* | CCND1 *DNA* | CCND1 *mRNA* |
|---|---|---|---|---|---|---|
| obr | D | − | N | + + + | N | + + + |
| ggu | D | − | N | + | N | + + |
| emh | D | − | N | + + | N | + |
| asn | D | − | N | + + + | N | (+) |
| sra | D | − | N | + + | N | + +(+) |
| kwa | D | − | N | + + | N | + + + |
| mob | D | − | N | + + | N | + + + |
| hso | M | ND | N | ND | N | ND |
| nak | N | + | A | + + + | N | + + |
| LOX[c] | D | − | N | + + | N | + +(+) |
| SESX[c] | D | − | N | + + | N | + + |
| No. of tumours with DNA alterations[d] | 9 | | 1 | | 0 | |

[a]N, normal; D, deletion; A, amplification; M, point mutation; ND, not determined. [b]Expression levels as described in Materials and methods. [c]Human melanoma cell lines. [d]Total number of tumours analysed = 61.

**Figure 1** Representative Southern blot analysis demonstrating the DNA status of *CDKN2*, *CDK4* and *CCND1* in tissue obtained from human metastatic melanomas (aga, kcl, lae, obr, ggu), from melanoma cell lines (LOX, THX) and from human leucocytes. The DNA (7 $\mu$g in each lane), digested with *Hin*dIII, was subsequently hybridised with probes encoding the three different genes and, as a control, with an *APOB* probe. Two additional bands emerged in the kcl tumour when hybridising with the *APOB* probe, probably due to a polymorphic site. Samples with homozygous deletion of *CDKN2*, scored as described in Materials and methods, are indicated by a D (deletion) below the corresponding panel.

**Figure 2** Direct sequencing of the *CDKN2* gene. The left panel shows the sequence observed in the hso tumour harbouring a mutation in codon 151 (CCC→TCC), while the right panel demonstrates the normal sequence.

**Figure 3** Northern blot analysis demonstrating the mRNA levels of *CDKN2*, *CDK4* and *CCND1*, for the same cases as listed in Figure 1, except that lane 8 contains RNA from a benign naevus. Total RNA (5 $\mu$g) applied in each lane was subsequently hybridised with probes encoding the three genes, and with an 18S rRNA probe as a control.

In general, the melanomas demonstrated low levels of *CDKN2* mRNA (Figure 3). Altogether 30 of the tumours (59%) lacked detectable expression of the inhibitor gene, as also was the case in benign naevi, basal cell carcinomas and normal skin tissue. Twenty-four of the tumours with no detectable *CDKN2* expression showed high transcript levels of either the kinase, *CDK4*, or the kinase activator, *CCND1*. As expected, none of the samples with homozygous deletion of *CDKN2* expressed the gene, whereas all seven cases showed high mRNA levels of either of the other genes (Table I). The three tumours containing a polymorphic site in *CDKN2* demonstrated low/undetectable levels of the corresponding mRNA (exemplified by sample aga in Figure 3). RNA was not available from the tumour with mutation in codon 151. Only nine samples (eight tumours and one cell line) showed a high expression of the gene (Table II and Figure 3). Of the eight tumours with high expression, four were characterised as superficial (4/25 = 16%), two as nodular (2/13 = 15%) and two were from other subgroups. The THX cell line demonstrated a very high level of *CDKN2* mRNA (Figure 3), and also a similarly elevated transcript level of the two other genes as was observed in the other cell lines.

The *CDK4* mRNA levels were high (+ + or + + +) in 39 of the melanoma patients (39/51 = 76%) (Table II) and in 28 of these the high kinase expression was accompanied by elevated levels of cyclin D1. The sample with *CDK4* amplification expressed a high level of the corresponding mRNA (Table I). Normal skin and benign naevi expressed low, but detectable, amounts of *CDK4* mRNA, whereas all cell lines showed high transcript levels of both the kinase and the activator.

High amounts of cyclin D1 mRNA were observed in 32 of the malignant melanomas (32/51 = 63%) (Table II). In comparison, four of five benign naevi had low, but detectable transcript levels, whereas lung and skin were the only normal tissues that showed high amounts of the mRNA.

**Table II** Tumours with high mRNA expression of *CDKN2*, *CDK4* and *CCND1*

| *Source of sample* | *No. analysed* | *No. of cases with high (++/+++) mRNA levels*[a] CDKN2 | CDK4 | CCND1 |
|---|---|---|---|---|
| Tumour tissue | 51 | 8 (16%) | 39 (76%) | 32 (63%) |
| Cell lines | 5 | 1 (20%) | 5 (100%) | 5 (100%) |
| Normal tissues | 12 | 0 | 4[b] | 2[c] |

[a]Expression levels as scored in Materials and methods. [b]High expression of *CDK4* in kidney, lung, ovary and breast gland. [c]High expression of *CCND1* in lung and skin.

**Figure 4** Western blot demonstrating pRb expression in human melanoma cell lines LOX (homozygous deletion of *CDKN2*) and THX (high *CDKN2* mRNA levels).

*Protein expression of pRb*

For immunohistochemical detection of pRb, frozen tissue sections were available from 15 of the melanoma metastases, and from 42 additional patients we obtained formalin-fixed paraffin-embedded sections. Heterogeneous pRb nuclear staining was identified in all samples, and the tumours were thus classified as pRb-positive. The phosphorylation status of the protein has not been examined in the patient material, but it is not inconceivable that the few patients showing high levels of the kinase inhibitor simultaneously express the phosphorylated form of the Rb protein.

The melanoma cells lines, however, were examined by Western blot analysis in order to determine the pRb status. The THX cell line, showing remarkably high expression of the *CDKN2* mRNA (Figure 3), expressed small/undetectable amounts of pRb (Figure 4), also confirmed by immunohistochemical detection on cytospins (results not shown). In contrast, the four other cell lines examined, two demonstrating homozygous loss of *CDKN2*, all expressed both the activated and the inactivated form of the Rb protein (exemplifed by the LOX cell line in Figure 4).

### Discussion

The present study of 61 biopsied metastases from sporadic melanoma metastases revealed abnormalities in the *CDKN2* gene in 13% of the cases. Thus, seven patients had homozygous deletions of the gene and one patient had a point mutation in codon 151. This frequency is in agreement with what has been reported previously on homozygous deletions in uncultured material from other tumour types (Cairns *et al.*, 1994*b*; Spruck *et al.*, 1994). Moreover, that only one point mutation was detected in our material is in agreement with what Ohta *et al.* (1994) reported. This frequency is, however, somewhat lower than that observed by Gruis *et al.* (1995), and furthermore, far lower than has been reported for cell lines. Also, in a total of five human melanoma cell lines examined we found no point mutations, but two homozygous deletions. Notably, our analysis demonstrated that homozygous deletion seems to be the predominant mechanism for inactivating the *CDKN2* gene in tumour biopsies, as has been reported for most tumour cell lines, including melanoma lines (Kamb *et al.*, 1994*a*; Gruis *et al.*, 1995; Liu *et al.*, 1995).

The low incidence of *CDKN2* deletions in our material compared with that observed in cell lines may be explained in part by methodological factors. Despite careful dissection of the tumour tissue before freezing, infiltration of normal cells in the tumour biopsy could result in an underestimation of the number of homozygous deletions observed in the melanoma patients. Southern blot analysis, however, as used here to detect homozygous deletions, appears to be somewhat less sensitive to a contribution from a moderate number of normal cells in the DNA preparation compared with PCR-based techniques. The difference in deletion frequency observed in cell lines compared with tumour biopsies might conceivably be a result of *in vitro* cell cultivation, as homozygous loss of *CDKN2* may provide additional growth advantage to cells in culture.

The PCR-based CDGE analysis has been shown to detect mutations, visualised by SYBR green I staining, if present in at least 10% of the cells when analysing homoduplex and down to 1% when analysing heteroduplex separation (Børresen, 1996). This indicates that it is unlikely that contaminating normal cell DNA should prevent the detection of mutant DNA. Our mutation analysis covers approximately 85% of the coding sequence (codon 20–152), and the possibility therefore exists that some mutations may not have been detected in the 5′-end of the gene. It should be noted, however, that by examining a mutation spectrum of 72 reported *CDKN2* mutations detected in different types of cancer, it was found, in contrast to that reported for tumour cell lines (Liu *et al.*, 1995), that the N-terminus seems to be less exposed to mutations (B Smith-Sørensen, personal communication). In fact, all tumour-associated *CDKN2* mutations so far reported are included in the sequences screened in this study.

In contrast to the mutation spectrum of *CDKN2* in non-skin malignancies, a remarkably high frequency of typical UV-induced CC→TT transitions has been observed in melanoma cell lines, reflecting a role of ultraviolet radiation in the generation of sporadic melanoma (Liu *et al.*, 1995; Pollock *et al.*, 1995). It is conceivable that UV-induced mutations may be selected for during *in vitro* cultivation. Furthermore, the codon 151 mutation observed in the hso tumour biopsy also falls into this group of mutations. Interestingly, the tumour named hso with mutated *CDKN2* was previously shown to harbour a mutated *TP53* gene (Flørenes *et al.*, 1994), but the TGT→TGG transversion in codon 275 of *TP53* is probably not induced by UV radiation. Several reports have demonstrated tumour samples harbouring mutations in both these genes, suggesting that the product of the tumour-suppressor genes functions in distinct pathways (Gruis *et al.*, 1995; Aagaard *et al.*, 1995).

It is important to regard p16 as a member of a cell cycle regulatory pathway involving at least three other gene products, namely: pRb, cdk4 and cyclin D1. pRb is the main mediator of growth suppression in this pathway, and the tumour-promoting effects of aberrations involving p16, cdk4 and cyclin D1 are most likely due to influence on the phosphorylation of the retinoblastoma protein. This mechanism for functional inactivation of pRb can involve increased kinase activity, caused either by loss of p16 inhibitor function or by overexpression of the kinase or the cyclin D1 activator. p16 is suggested to functional as a negative regulator of cdk4 activity once pRb has been inactivated by phosphorylation, and p16-induced growth suppression has been demonstrated in cells containing a functional pRb (Koh *et al.*, 1995; Lukas *et al.*, 1995; Medema *et al.*, 1995; Serrano *et al.*, 1995).

Moreover, in cells harbouring a constitutively inactivated Rb protein, elevated p16 expression concomitant with inhibition of the kinase activity has also been detected (Serrano *et al.*, 1993). However, in such cells overexpression of p16 does not induce cell cycle arrest, probably due to the fact that Rb represents the major target protein and is required as a downstream effector in p16-mediated growth arrest (Koh *et al.*, 1995; Lukas *et al.*, 1995; Medema *et al.*, 1995; Serrano *et al.*, 1995).

In agreement with other studies (Serrano *et al.*, 1993; Li *et al.*, 1994; Tam *et al.*, 1994*a*; Yeager *et al.*, 1995) showing correlation between high expression of p16 and absence of functional Rb protein, we found in our panel of metastatic melanomas only nine tumours (17%) with high levels of p16 mRNA. All the examined cases expressed the Rb protein as detected by immunohistochemistry, a finding consistent with the low frequency of pRb deficiency observed in melanomas (Horowitz *et al.*, 1990; Lewis *et al.*, 1993). It is conceivable that the Rb protein might be inactivated by phosphorylation in the few tumours showing high inhibitor expression. Among the cell lines, high levels of both phosphorylated and unphosphorylated pRb were detected in those lacking *CDKN2*, whereas in one cell line overexpression of *CDKN2* mRNA was accompanied with low or nearly undetectable levels of pRb.

That a high fraction of metastatic melanomas did not express detectable levels of *CDKN2* mRNA (60%) is in agreement with recent results (Reed *et al.*, 1995), demonstrating loss of p16 protein expression in metastatic lesions (44%), but not in primary melanomas, melanomas *in situ* and atypical naevi. The mechanism by which loss of p16 expression, without homozygous deletion, is associated with invasiveness and progression of malignant melanomas is still unclear. Functional inactivation of the *CDKN2* gene by hypermethylation of the 5′ CpG island has been demonstrated in cell lines and biopsies of non-small-cell lung carcinomas (Merlo *et al.*, 1995; Otterson *et al.*, 1995). Whether *de novo* methylation plays a role in controlling the expression of this gene in sporadic melanoma lesions has not yet been examined.

Notably, the melanomas showed very high levels of *CDK4* mRNA, often accompanied with high expression of cyclin D1. Amplification of both these genes has been found in different cancer types (Lammie *et al.*, 1991; Khatib *et al.*, 1993; Mælandsmo *et al.*, 1995), but seems to be a rare event in the development of metastatic malignant melanomas, as no *CCND1* amplification and only one *CDK4* amplification was observed. Tumour-associated overexpression of cyclin D1, without any observed amplification of the gene, has previously been reported in primary malignant melanomas (Bartkova *et al.*, 1995). Recently, a somatic point mutation in the *CDK4* gene was demonstrated in a human melanoma, disrupting the interaction between the kinase and the p16 inhibitor (Wölfel *et al.*, 1995). It is believed that such mutations may constitute a new mechanism to inactivate this regulatory pathway in some tumour cells. Moreover, He *et al.* (1994) suggested overexpression of cdk4 in excess of what can be inactivated by p16, as an alternative mechanism for suppressing pRb growth control in glioma cell lines. However, the relative abundance of D-type cyclins and p16 determine the cdk4 activity, and it has been demonstrated that synthesis of cyclin D1, a protein with short half-life, is necessary and rate-limiting for G1 progression (Matsushime *et al.*, 1991; Baldin *et al.*, 1993). According to this, it has been postulated that tumour cells can obtain a growth advantage from a moderate overexpression of D-type cyclins and/or from a more massive overexpression of the catalytic subunit (Tam *et al.*, 1994*b*). That all the melanoma biopsies were from metastases, often aggressive tumours with high proliferation rates, may provide a reasonable explanation for the observed high expression levels. However, it should be noted that no significant association between high cyclin D1 expression and prognostic factors, such as relapse-free period and tumour thickness, was revealed (not shown).

Several recent reports suggest that disturbance of a single growth regulatory pathway, resulting in an inactivation of pRb function, is of significant importance in the development of different types of cancer. Alterations of any of the components in the pathway constituted by p16, pRb, cyclin D1 and cdk4 may be sufficient to create an imbalance in the system, thereby providing the cells with a growth advantage. Although the frequency of *CDKN2* gene aberrations was found to be low in our panel of malignant melanomas, we observed a high number of metastatic lesions without *CDKN2* mRNA expression together with overexpression of *CDK4* and *CCND1*. Altogether the results may indicate that functional inactivation of pRb is involved in the tumorigenesis of sporadic, malignant melanomas.

### Abbreviations

*CDK4*, cyclin-dependent kinase 4; *CDKN2*, the cdk4 inhibitor, p16; *CCND1*, cyclin D1; pRb, the retinoblastoma protein; *APOB*, apolipoprotein B; SDS, sodium dodecyl sulphate; SSC, standard saline citrate; PCR, polymerase chain reaction; CDGE, constant denaturant gel electrophoresis.

### Acknowledgements

We thank Frode Kristiansen, Gunn Elin Trones, Turid Mellingsæter, Siri Juell, Anne Forus, Ellen Hellesylt, Mette Myre and Hilde Johnsen for excellent technical assistance, Birgitte Smith-Sørensen for generating the p16 mutation spectrum and Frances Jaques for her secretarial assistance. This work was supported by The Norwegian Cancer Society and the Anders Jahre Foundation.

### References

AAGAARD L, LUKAS J, BARTKOVA J, KJERULFF AA, STRAUSS M AND BARTEK J. (1995). Aberrations of p16$^{Ink4}$ and retinoblastoma tumour-suppressor genes occur in distinct sub-sets of human cancer cell lines. *Int. J. Cancer*, **61**, 115–120.

BALDIN V, LUKAS J, MARCOTE M, PAGANO M AND DRAETTA G. (1993). Cyclin D1 is a nuclear protein required for cell cycle progression in G1. *Genes Dev.*, **7**, 812–821.

BARTKOVA J, LUKAS J, STRAUSS M AND BARTEK J. (1995). Cyclin D1 oncoprotein aberrantly accumulates in malignancies of diverse histogenesis. *Oncogene*, **10**, 775–778.

BØRRESEN AL. (1996). Constant denaturant gel electrophoresis (CDGE) in mutation screening. In *Technologies for Detection of DNA Damage and Mutations*. Pfeifer GP (ed). Plenum Press (in press).

BUCKLEY MF, SWEENEY KJ, HAMILTON JA, SINI RL, MANNING DL, NICHOLSON RI, DEFAZIO A, WATTS CK, MUSGROVE EA AND SUTHERLAND RL. (1993). Expression and amplification of cyclin genes in human breast cancer. *Oncogene*, **8**, 2127–2133.

CAIRNS P, MAO L, MERLO A, LEE DJ, SCHWAB D, EBY Y, TOKINO K, RIET PVD, BLAUGRUND JE AND SIDRANSKY D. (1994*a*). Rates of p16 (MTS1) mutations in primary tumors with 9p loss. *Science*, **265**, 415–416.

CAIRNS P, TOKINO K, EBY Y AND SIDRANSKY D. (1994*b*). Homozygous deletions of 9p21 in primary human bladder tumors detected by comparative multiplex polymerase chain reaction. *Cancer Res.*, **54**, 1422–1424.

CANNON-ALBRIGHT LA, GOLDGAR DE, MEYER LJ, LEWIS CM, ANDERSON DE, FOUNTAIN JW, HEGI ME, WISEMAN RW, PETTY EM, BALE AE, OLOPADE OI, DIAZ MO, KWIATKOWSKI D, PIEPKORN MW, ZONE JJ AND SKOLNICK MH. (1992). Assignment of a locus for familial melanoma, MLM, to chromosome 9p13-p22. *Science*, **258**, 1148–1152.

CATTORETTI G, BECKER MH, KEY G, DUCHROW M, SCHLUTER C, GALLE J AND GERDES J. (1992). Monoclonal antibodies against recombinant parts of the Ki-67 antigen (MIB 1 and MIB 3) detect proliferating cells in microwave-processed formalin-fixed paraffin sections. *J. Pathol.*, **168**, 357–363.

CHURCH GM AND GILBERT W. (1984). Genomic sequencing. *Proc. Natl Acad. Sci. USA*, **81**, 1991–1995.

FEINBERG AP AND VOGELSTEIN B. (1983). A technique for radio labelling DNA restriction endonuclease fragments to high specific activity. *Anal. Biochem.*, **132**, 6–13.

FLØRENES VA, ØYJORD T, HOLM R, SKREDE M, BØRRESEN A-L, NESLAND JM AND FODSTAD Ø. (1994). *TP53* allele loss, mutations and expression in malignant melanoma. *Br. J. Cancer*, **69**, 253–259.

FORUS A, FLØRENES V, MÆLANDSMO GM, FODSTAD Ø AND MYKLEBOST O. (1994). 12q13-14 amplica in human sarcomas without *MDM2* include *CDK4*, *SAS* and *GADD153/CHOP*. *Cancer Genet. Cytogenet.*, **77**, 200.

FOUNTAIN JW, KARAYIORGOU M, ERNSTOFF MS, KIRKWOOD JM, VLOCK DR, TITUS-ERNSTOFF L, BOUCHARD B, VIJAYA-SARADHI S, HOUGHTON AN, LAHTI J, KIDD VJ, HOUSMAN DE AND DRACOPOLI NC. (1992). Homozygous deletions within human chromosome band 9p21 in melanoma. *Proc. Natl.Acad. Sci. USA*, **89**, 10557–10561.

GOODRICH DW AND LEE WH. (1993). Molecular characterization of the retinoblastoma susceptibility gene. *Biochim. Biophys. Acta*, **1155**, 43–61.

GRUIS NA, WEAVER-FELDHAUS J, LIU Q, FRYE C, EELES R, ORLOW I, LACOMBE L, PONCE-CASTANEDA V, LIANES P, LATRES E, SKOLNICK M, CORDON-CARDO C AND KAMB A. (1995). Genetic evidence in melanoma and bladder cancers that p16 and p53 function in separate pathways of tumor suppression. *Am. J. Pathol.*, **146**, 1199–1206.

HE J, ALLEN JR, COLLINS VP, ALLALUNIS-TURNER MJ, GODBOUT R, DAY RSR III AND JAMES CD. (1994). *CDK4* amplification is an alternative mechanism to p16 gene homozygous deletion in glioma cell lines. *Cancer Res.*, **54**, 5804–5807.

HINDS PW, DOWDY SF, EATON EN, ARNOLD A AND WEINBERG RA. (1994). Function of a human cyclic gene as an oncogene. *Proc. Natl Acad. Sci. USA*, **91**, 709–713.

HOROWITZ JM, PARK SH, BOGENMANN E, CHENG JC, YANDELL DW, KAYE FJ, MINNA JD, DRYJA TP AND WEINBERG RA. (1990). Frequent inactivation of the retinoblastoma anti-oncogene is restricted to a subset of human tumor cells. *Proc. Natl. Acad. Sci. USA*, **87**, 2775–2779.

HOVIG E, SMITH-SØRENSEN B, BRØGGER A AND BØRRESEN AL. (1991). Constant denaturant gel electrophoresis, a modification of denaturing gradiant gel electrophoresis, in mutation detection. *Mutat. Res.*, **262**, 63–71.

HSU SM, RAINE L AND FANGER H. (1981). A comparative study of the peroxidase–antiperoxidase method and an avidin–biotin complex method for studying polypeptide hormones with radioimmunoassay antibodies. *Am. J. Clin. Pathol.*, **75**, 734–738.

HUNTER T AND PINES J. (1994). Cyclins and Cancer II: Cyclin D and CDK inhibitors come of age. *Cell*, **79**, 573–582.

HUSSUSIAN CJ, STRUEWING JP, GOLDSTEIN AM, HIGGINS PA, ALLY DS, SHEAHAN MD, CLARK WH JR, TUCKER MA AND DRACOPOLI NC. (1994). Germline p16 mutations in familial melanoma. *Nature Genet.*, **8**, 15–21.

JIANG W, KAHN SM, TOMITA N, ZHANG YJ, LU SH AND WEINSTEIN IB. (1992). Amplification and expression of human cyclin D gene in esophageal cancer. *Cancer Res.*, **52**, 2980–2983.

KAMB A. (1995). Cell-cycle regulators and cancer. *Trends Genet.*, **11**, 136–140.

KAMB A, GRUIS NA, WEAVER-FELDHAUS J, LIU Q, HARSHMAN K, TAVTIGIAN SV, STOCKERT E, DAY RR, JOHNSON BE AND SKOLNICK MH. (1994*a*). A cell cycle regulator potentially involved in genesis of many tumor types. *Science*, **264**, 436–440.

KAMB A, SHATTUCK-EIDENS D, EELES R, LIU Q, GRUIS NA, DING W, HUSSEY C, TRAN T, MIKI Y, WEAVER-FELDHAUS J, McCLURE M, AITKEN JF, ANDERSON DE, BERGMAN W, FRANTS R, GOLDGAR DE, GREEN A, MacCLENNAN R, MARTIN NG, MEYER LJ, YOUL P, ZONE JJ, SKOLNICK MH AND CANNON-ALBRIGHT LA. (1994*b*). Analysis of the p16 gene (*CDKN2*) as a candidate for the chromosome 9p melanoma susceptibility locus. *Nature Genet.*, **8**, 23–26.

KHATIB ZA, MATSUSHIME H, VALENTINE M, SHAPIRO DN, SHERR CJ AND LOOK AT. (1993). Coamplification of the *CDK4* gene with *MDM2* and *GLI* in human sarcomas. *Cancer Res.*, **53**, 5535–5541.

KOH J, ENDERS GH, DYNLACHT BD AND HARLOW E. (1995). Tumour-derived p16 alleles encoding proteins defective in cell-cycle inhibition. *Nature*, **375**, 506–510.

LAMMIE GA, FANTL V, SMITH R, SCHUURING E, BROOKES S, MICHALIDES R, DICKSON C, ARNOLD A AND PETERS G. (1991). *D11S287*, a putative oncogene on chromosome 11q13, is amplified and expressed in squamous cell and mammary carcinomas and linked to *BCL-1*. *Oncogene*, **6**, 439–444.

LEWIS DC, WARREN N, SHUKLA VK, GRIMSHAW D, LAIDLER P AND PADUA RA. (1993). Gross rearrangements and deletions of the retinoblastoma gene are rare in malignant melanoma. *Acta Derm. Venereol.*, **73**, 236.

LI Y, NICHOLS MA, SHAY JW AND XIONG Y. (1994). Transcriptional repression of the D-type cyclin-dependent kinase inhibitor p16 by the retinoblastoma susceptibility gene product pRb. *Cancer Res.*, **54**, 6078–6082.

LIU Q, NEUHAUSEN S, McCLURE M, FRYE C, WEAVER-FELDHAUS J, GRUIS NA, EDDINGTON K, ALLALUNIS-TURNER MJ, SKOLNICK MH, FUJIMURA FK AND KAMB A. (1995). *CDNK2* (*MTS1*) tumor suppressor gene mutations in human tumor cell lines. *Oncogene*, **10**, 1061–1067.

LUKAS J, PARRY D, AAGAARD L, MANN DJ, BARTKOVA J, STRAUSS M, PETERS G AND BARTEK J. (1995). Retinoblastoma-protein-dependent cell-cycle inhibition by the tumour suppressor p16. *Nature*, **375**, 503–506.

MÆLANDSMO GM, BERNER JM, FLØRENES VA, FORUS A, HOVIG E, FODSTAD Ø AND MYKLEBOST O. (1995). Homozygous deletion frequency and expression levels of the *CDKN2* gene in human sarcomas – relationship to amplification and mRNA levels of *CDK4* and *CCND1*. *Br. J. Cancer*, **72**, 393–398.

MANIATIS T, FRITSCH EF AND SAMBROOK J. (1982). *Molecular Cloning: a Laboratory Manual*. Cold Spring Harbor Laboratory Press: Cold Spring Harbor, NY.

MATSUSHIME H, ROUSSEL RA, ASHMUN RA AND SHERR CJ. (1991). Colony-stimulating factor 1 regulates novel cyclins during the G1 phase of the cell cycle. *Cell*, **65**, 701–713.

MEDEMA RH, HERRERA RE, LAM F AND WEINBERG RA. (1995). Growth suppression by p16$^{Ink4}$ requires functional retinoblastoma protein. *Proc. Natl Acad. Sci. USA*, **92**, 6289–6293.

MERLO A, HERMAN JG, MAO L, LEE DJ, GABRIELSON E, BURGER PC, BAYLIN SB AND SIDRANSKY D. (1995). 5′ CpG island methylation is associated with transcriptional silencing of the tumour suppressor p16/*CDKN2/MTS1* in human cancers. *Nature Med.*, **1**, 686–692.

MOTOKURA T AND ARNOLD A. (1993). Cyclin D and oncogenesis. *Curr. Opin. Genet. Dev.*, **3**, 5–10.

NOBORI T, MIURA K, WU DJ, LOIS A, TAKABAYASHI K AND CARSON DA. (1994). Deletions of the cyclin-dependent kinase-4 inhibitor gene in multiple human cancers. *Nature*, **368**, 753–756.

OHTA M, NAGAI H, SHIMIZU M, RASIO D, BERD D, MASTRANGELO M, SINGH AD, SHIELDS JA, SHIELDS CL, CROCE CM AND HUEBNER K. (1994). Rarity of somatic and germline mutations of the cyclin-dependent kinase 4 inhibitor gene, *CDK4I*, in melanoma. *Cancer Res.*, **54**, 5269–5272.

OTTERSON GA, KHLEIF SN, CHEN W, COXON AB AND KAYE FJ. (1995). *CDKN2* gene silencing in lung cancer by DNA hypermethylation and kinetics of p16$^{INK4}$ protein induction by 5-aza 2′ deoxycytidine. *Oncogene*, **11**, 1211–1216.

POLLOCK PM, YU F, QIU L, PARSONS PG AND HAYWARD NK. (1995). Evidence for u.v. induction of *CDKN2* mutations in melanoma cell lines. *Oncogene*, **11**, 663–668.

RANADE K, HUSSUSSIAN CJ, SIKORSKI RS, VARMUS HE, GOLDSTEIN AM, TUCKER MA, SERRANO M, HANNON GJ, BEACH D AND DRACOPOLI NC. (1995). Mutations associated with familial melanoma impair p16$^{INK4}$ function. *Nature Genet.*, **10**, 114–116.

REED JA, LOGANZO JR F, SHEA CR, WALKER GJ, FLORES JF, GLENDENING JM, BOGDANY JK, SHIEL MJ, HALUSKA FG, FOUNTAIN JW AND ALBINO AP. (1995). Loss of expression of the p16/cyclin-dependent kinase inhibitor 2 tumor suppressor gene in melanocytic lesions correlates with invasive stage of tumor progression. *Cancer Res.*, **55**, 2713–2718.

REIFENBERGER G, REIFENBERGER J, ICHIMURA K, MELTZER PS AND COLLINS VP. (1994). Amplification of multiple genes from chromosomal region 12q13-14 in human malignant gliomas: preliminary mapping of the amplicons shows preferential involvement of *CDK4*, *SAS* and *MDM2*. *Cancer Res.*, **54**, 4299–4303.

SERRANO M, HANNON GJ AND BEACH D. (1993). A new regulatory motif in cell-cycle control causing specific inhibition of cyclin D/CDK4. *Nature*, **366**, 704–707.

Serrano M, Gomez-Lahoz E, DePinho RA, Beach D and Bar-Sagi D. (1995). Inhibition of ras-induced proliferation and cellular transformation by $p16^{INK4}$. *Science*, **267**, 249–252.

Spruck III CH, Gonzalez-Zulueta M, Shibata A, Simoneau AR, Lin M-F, Gonzales F, Tsai YC and Jones PA. (1994). p16 gene in uncultured tumours. *Nature*, **370**, 183–184.

Sun Y, Hildesheim A, Lanier AE, Cao Y, Yao KT, Raab-Traub N and Yang CS. (1995). No point mutation but decreased expression of the p16/*MTS1* tumor suppressor gene in nasopharyngeal carcinomas. *Oncogene*, **10**, 785–788.

Tam SW, Shay JW and Pagano M. (1994*a*). Differential expression and cell cycle regulation of the cyclin-dependent kinase 4 inhibitor $p16^{INK4}$. *Cancer Res.*, **54**, 5816–5820.

Tam SW, Theodoras AM, Shay JW, Draetta GF and Pagano M. (1994*b*). Differential expression and regulation of cyclin D1 protein in normal and tumor human cells: association with Cdk4 is required for Cyclin D1 function in G1 progression. *Oncogene*, **9**, 2663–2674.

Wadayama B, Toguchida J, Shimizu T, Ishizaki K, Sasaki MS, Kotoura Y and Yamamuro T. (1994). Mutation spectrum of the retinoblastoma gene in osteosarcomas. *Cancer Res.*, **54**, 3042–3048.

Weinberg RA. (1995). The retinoblastoma protein and cell cycle control. *Cell*, **81**, 323–330.

Wölfel T, Hauer M, Schneider J, Serrano M, Wölfel C, Klehmann-Hieb E, de Plaen E, Hankeln T, Meyer zum Büschenfelde K-H and Beach D. (1995). A $p16^{INK4a}$-insensitive *CDK4* mutant targeted by cytolytic T lymphocytes in a human melanoma. *Science*, **269**, 1281–1284.

Xu HJ, Hu SX, Cagle PT, Moore GE and Benedict WF. (1991). Absence of retinoblastoma protein expression in primary non-small cell lung carcinomas. *Cancer Res.*, **51**, 2735–2739.

Yeager T, Stadler W, Belair C, Puthenveettil J, Olopade O and Reznikoff C. (1995). Increased p16 levels correlate with pRb alterations in human urothelial cells. *Cancer Res.*, **55**, 493–497.